# Functional and Radiological Improvement in a COVID-19 Pneumonia Patient Treated With Steroids

**DOI:** 10.7759/cureus.15257

**Published:** 2021-05-26

**Authors:** Rahul Singh, Dominic Gaziano

**Affiliations:** 1 Accident and Emergency, Dr. Balwant Singh's Hospital, Georgetown, GUY; 2 Family Medicine, Amita Health, Chicago, USA

**Keywords:** corona virus disease 2019, corticosteroid medication, covid induced ards, covid, long covid, ards (acute respiratory distress syndrome), sars-cov-2 induced ards, acute respiratory distress syndrome [ards]

## Abstract

Like its predecessors, coronavirus disease 2019 (COVID-19) can lead to long-term health-related consequences in a significant segment of the afflicted population. Although the medical community has developed multiple vaccines by now, COVID-19 has affected over 100 million individuals worldwide and will infect millions more before vaccines can be effectively distributed on a global scale. Additionally, it seems probable that another outbreak caused by a coronavirus may occur in the future, given that this is the third outbreak caused by a coronavirus in recent history. In light of this, the medical community must develop reliable methods of curtailing long-term sequelae of coronaviruses, and the use of corticosteroids in affected patients may be vital for this purpose. In this report, we present a case of progressive dyspnea caused by COVID-19 pneumonia; the patient was treated with a short course of oral corticosteroids, and subsequently showed marked improvement of the dyspnea with corresponding improvements on chest CT.

## Introduction

Coronavirus disease 2019 (COVID-19), caused by severe acute respiratory syndrome coronavirus 2 (SARS-CoV-2), has already affected 100 million people worldwide. While we are not fully familiar with the long-term complications of COVID-19, the emerging data is alarming. One study has shown that at the 60-day follow-up of patients discharged from a hospital after recovery from COVID-19, 87% of the patients reported experiencing persistent symptoms, and 44% of them reported a worsened quality of life [1]. The most common symptoms reported were fatigue and dyspnea [1], with the latter likely corresponding to the degree of radiographically detectable pulmonary fibrotic changes. These authors have expressed the fear that even after the release of a vaccine, for years to come, medical practitioners worldwide will still be picking up the pieces in the wake of the 21st century’s first global pandemic.

In this report, we discuss a case of a male patient with progressive dyspnea caused by COVID-19 pneumonia, who was treated with a short course of oral corticosteroids; he subsequently showed marked improvement of his dyspnea with corresponding radiological improvements on chest CT.

## Case presentation

A 65-year-old male presented to our emergency department due to three days of increasing shortness of breath, and chest pain. The patient had been discharged from another hospital nine days before presenting to us, where he had been admitted for 22 days for viral pneumonia due to COVID-19. During the previous admission, he had required oxygen supplementation at the rate of 3 L/min via nasal cannula (NC). He had continued to use this even after discharge and had been unable to wean himself off.

The patient had been experiencing increasing shortness of breath over the last three days, while his chest pain was midsternal, pleuritic, and aggravated by deep inspiration and coughing. Multiple family members living with the patient had tested positive for COVID-19, but they had since recovered. At the time of presentation, the patient also had an intermittent dry cough. 

Upon examination in the emergency department, the patient was found to be able to speak in complete sentences; he was also afebrile, tachycardic (138 beats per minute), and tachypneic (38 breaths per minute). It was noted that the patient’s tachypnea tended to worsen upon exertion. The patient was admitted to the hospital’s critical care unit (CCU).

Investigations

**Figure 1:**
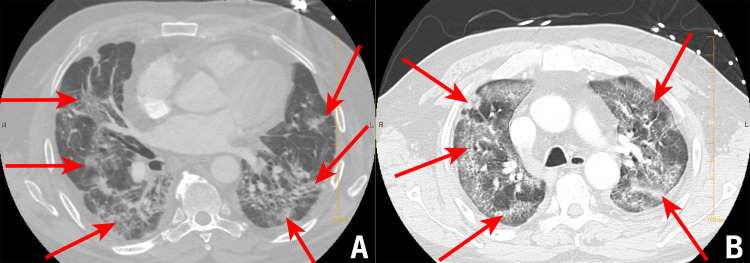
CT angiogram performed at the time of admission A and B: CT angiogram demonstrating fibrotic changes with reticulations, traction bronchiectasis, and areas of "crazy paving" ground-glass opacities consistent with sequelae of COVID-19 pneumonia CT: computed tomography; COVID-19: coronavirus disease 2019

A CT angiogram showed no evidence of pulmonary embolism but revealed fibrotic changes with reticulations, traction bronchiectasis, and areas of "crazy paving" ground-glass opacities (Figure 1A). Transthoracic echocardiogram (TTE) showed a normal ejection fraction, while his laboratory tests were remarkable for an elevated plasma D-dimer of 1,040 FEU, and an elevated interleukin 6 (IL-6) of 34.6 pg/ml. Of note, the patient’s procalcitonin was low (<0.20 ng/mL), while his white cell count and troponin were within normal ranges.

While the patient had previously tested positive for COVID-19, more than 30 days before presenting to us, a repeat nasopharyngeal swab performed at the time of admission was negative for SARS-CoV-2. Later, a serological test for the SARS-CoV-2 immunoglobulin G (IgG) antibody returned positive. The patient underwent an electrocardiogram (EKG), which showed sinus tachycardia. Saturation testing was performed on three days during his admission, including the date of discharge (Table 1).

**Table 1:** Saturation testing performed during the patient's admission NA: nasal cannula

	Day 1	Day 2	Day 3
Testing at rest with 3 L/min via NC	96%	98%	98%
Testing on room air with exertion	83%	89%	86%
Testing with exertion on 1 L/min	88%	90%	90%
Testing with exertion on 2 L/min	91%	92%	91%
Testing with exertion on 3 L/min	93%	N/A	93%

Differential diagnosis

At the time of presentation, the patient’s chest pain and dyspnea raised concerns for pulmonary embolism; however, this was considered unlikely following the CT angiogram of his thorax and TTE. The patient’s CT lung findings of ground-glass opacities, and traction bronchiectasis, raised suspicions of cryptogenic organizing pneumonia (COP). However, given that the patient had been discharged from another hospital nine days prior to presenting to us, where he had been admitted due to pneumonia caused by SARS-CoV-2, COP was deemed less probable than pneumonia caused by SARS-CoV-2. Community-acquired pneumonia was also considered, although this was also determined to be unlikely since the patient was afebrile, had a low procalcitonin level, and a normal white cell count. The patient’s differential diagnosis also included SARS-CoV-2-related cardiomyopathy, but this was also considered improbable following a normal EKG and TTE.

Treatment

At the emergency department, the patient was placed on 3 L/min of supplemental oxygen via NC. Following an evaluation, and an estimation that he was at moderate risk of respiratory decompensation, he was admitted to our hospital's CCU. The day after admission, he was started on oral prednisone 40 mg for two weeks. Following a home oxygen evaluation, it was determined that the patient would require 3 L/min of supplemental oxygen via NC, which he was discharged with; he was also advised to perform six weeks of cardiovascular exercises to help improve his breathing.

Outcome and follow-up

After being placed on 3 L/min of supplemental oxygen via NC, the patient became more comfortable; his tachypnea initially persisted but improved by the second day. On the third day of admission, since he was found to be oxygenating better, he was transferred from the CCU to the ward, but the patient would still become short of breath while walking to the bathroom. Five days after presenting to us, the patient was discharged with 3 L/min of supplemental oxygen via NC. He was given his complete course of oral prednisone, advised to do six weeks of cardiovascular exercises to help improve his breathing, and asked to appear for a follow-up in two weeks.

During his first follow-up appointment two weeks after discharge, the patient was found to have had remarkable resolution of his dyspnea, even with exertion. He was able to go through the whole day without the use of supplemental oxygen. While the chest pain was still present, it had decreased since the time of his presentation to us and was no longer aggravated by inspiration.

**Figure 2:**
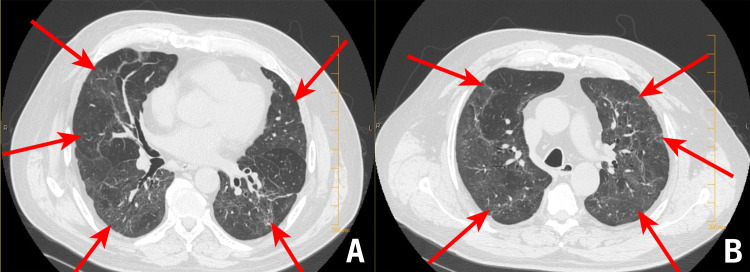
Follow-up CT scan performed six weeks after discharge A and B: images from the follow-up CT scan demonstrating significant resolution of the fibrotic changes present at the time of admission CT: computed tomography

At his six-week follow-up, the patient was found to have achieved a complete resolution of the shortness of breath. A second CT scan was performed two months after the patient's initial presentation (Figure 2). The findings of this scan showed a striking resolution of the fibrotic changes observed on the previous CT scan, such as the ground-glass opacities and traction bronchiectasis.

## Discussion

We have not yet gained a sufficient understanding of the long-term sequelae of COVID-19, primarily because of its relatively recent emergence. However, we have been able to draw some preliminary conclusions. Almost 90% of patients assessed at a two-month follow-up reported at least one persistent symptom [1]. Severe acute respiratory syndrome (SARS) and the Middle East respiratory syndrome (MERS) were the other two coronavirus-induced outbreaks in recent history, and both of these are associated with long-term functional and radiological sequelae [2-4]. Therefore, it is highly probable that healthcare systems worldwide will be managing patients experiencing sequelae of the COVID-19 outbreak for years to come, and it is imperative that a proper approach be developed to limit the sequelae of COVID-19.

At the time of our patient's initial presentation to us, a repeat nasopharyngeal swab was found to be negative for SARS-CoV-2. However, his CT findings of "crazy paving" ground-glass opacities were consistent with the sequelae of COVID-19 pneumonia [5]. Since our patient was already receiving oxygen, we administered corticosteroids, in accordance with the recommendations of the Randomized Evaluation of COVID-19 Therapy (RECOVERY) trial, which had found that the use of corticosteroids was associated with better outcomes among patients receiving respiratory support [6].

After considering the extent of the fibrotic changes on our patient’s initial CT scan, it was assumed that there would be proportional residual findings on follow-up, with corresponding functional abnormalities, as is consistent with acute respiratory distress syndrome (ARDS) [7]. However, follow-up imaging and encounters revealed an extraordinarily marked resolution of fibrotic lung lesions and a complete resolution of the patient's dyspnea. 

The fibrotic changes observed in the lungs of COVID-19 patients, such as ours, are thought to be due to an induced "cytokine storm" [8]. Elevated cytokines IL-6 and tumor necrosis factor-α (TNF-α) are particularly associated with worse outcomes in patients of COVID-19 [9], as well as in patients of ARDS due to causes unrelated to COVID-19 [10]. Moreover, ground-glass opacities, which are characteristic of COVID-19 [5] and observed in our patient, are compatible with the late, fibroproliferative phase of ARDS [10].

During the late, fibroproliferative phase of ARDS, the lung can undergo vast fibrotic changes as it attempts to recover from injury, with better outcomes being associated with the reversal of this fibroproliferation [10]. This is considered especially relevant to COVID-19 since the late, fibroproliferative phase of ARDS is thought to be responsive to corticosteroids [11], as these drugs can potentially lead to a reversal of fibroproliferation, a reduction in plasma levels of TNF-α and IL-6, and overall improved outcomes [10]. Of note, the administration of corticosteroids too early in ARDS may lead to increased lung damage [12]. These findings could explain why corticosteroids have not been found to benefit patients of COVID-19 if they are not on respiratory support [6], and why other studies have found that corticosteroids did not reduce mortality in the general population [13].

At the time of his discharge, we advised our patient to initiate cardiovascular exercises. The patient followed the medical teams' advice and followed an exercise routine focused on improving his cardiovascular health. Other studies have observed that muscle weakness due to deconditioning caused by prolonged bed-rest may have been a contributing factor in the functional respiratory impairment experienced by SARS patients [4]. Thus, we believe that the cardiovascular exercise routine may have helped alleviate the physical deconditioning, and played a role in the rapid resolution of dyspnea in our patient.

Other studies have noted significant improvement in pulmonary fibrotic changes in COVID-19 patients treated with corticosteroids [14-16]. The use of high-dose corticosteroids may have also restricted the incidence of fibrosis observed in SARS patients [3,4]. Due to the worldwide spread of COVID-19, with over 100 million confirmed cases at the time of writing, we believe it is imperative that more research be conducted to devise methods to attenuate the pulmonary sequelae in patients who have recovered from COVID-19. Based on our experience, corticosteroids and cardiovascular exercise possibly have a major role to play in attenuating long-term morbidity due to the COVID-19 outbreak.

## Conclusions

We discussed the case of a patient who presented with significant functional and radiological sequelae due to COVID-19, which significantly improved following a short course of corticosteroids. Similar to previous outbreaks caused by other coronaviruses, COVID-19 may be associated with significant long-term functional and radiological sequelae. Corticosteroids and cardiovascular exercise may play a crucial role in mitigating the potential long-term sequelae of COVID-19.
